# Enzyme-guided DNA Sewing Architecture

**DOI:** 10.1038/srep17722

**Published:** 2015-12-04

**Authors:** In Hyun Song, Seung Won Shin, Kyung Soo Park, Yves Lansac, Yun Hee Jang, Soong Ho Um

**Affiliations:** 1 School of Chemical Engineering, Sungkyunkwan University, Suwon, Gyeonggi-do, 440-746, South Korea; 2 SKKU Advanced Institute of Nanotechnology (SAINT), Sungkyunkwan University, Suwon, Gyeonggi-do, 440-746, South Korea; 3 GREMAN, UMR7347, Université François Rabelais, 37200, France; 4 School of Materials Science and Engineering, Gwangju Institute of Science and Technology, Gwangju, 61005, South Korea

## Abstract

With the advent of nanotechnology, a variety of nanoarchitectures with varied physicochemical properties have been designed. Owing to the unique characteristics, DNAs have been used as a functional building block for novel nanoarchitecture. In particular, a self-assembly of long DNA molecules via a piece DNA staple has been utilized to attain such constructs. However, it needs many talented prerequisites (e.g., complicated computer program) with fewer yields of products. In addition, it has many limitations to overcome: for instance, (i) thermal instability under moderate environments and (ii) restraint in size caused by the restricted length of scaffold strands. Alternatively, the enzymatic sewing linkage of short DNA blocks is simply designed into long DNA assemblies but it is more error-prone due to the undeveloped sequence data. Here, we present, for the first time, a comprehensive study for directly combining DNA structures into higher DNA sewing constructs through the 5′-end cohesive ligation of T4 enzyme. Inspired by these achievements, the synthesized DNA nanomaterials were also utilized for effective detection and real-time diagnosis of cancer-specific and cytosolic RNA markers. This generalized protocol for generic DNA sewing is expected to be useful in several DNA nanotechnology as well as any nucleic acid-related fields.

With the rapid growth of DNA nanotechnology, self-assembled DNA materials have been designed and manufactured^1^,^2^,^3^,^4^. Due to the precise controllability and intrinsic selective specificity, nucleic acids, i.e., DNA or RNA, can be used as generic nano-brick-and-mortar material. To date, several complexes of DNA origami have been achieved and include sphere, hexagon, tube, Mobius, and emoticon shapes^4^,^5^,^6^. Furthermore, such DNA can be practically used as a seed template of DNA crystal or as a therapeutic agent or its vehicle^7^. To construct these shapes, one methodology has been utilized: stapling of long and single DNA ropes^5^,^6^. However, it has suffered from several limitations to overcome: for instance, (i) thermal instability under moderate environment and (ii) restraint in size caused by the restricted length of scaffold strands^8^,^9^,^10^,^11^. Then, a new technique which is sewing individual DNA blocks via a enzymatic ligation process has been developed as an alternative^12^,^13^. In past, enzymatic interlinking of single DNA blocks was investigated for higher DNA structure^4^,^13^,^14^,^15^. It is not as frequently employed because it is more error-prone and results in imperfect DNA materials through accidental mismatch ligations, despite several advantages of this enzymatic interlinkage method (e.g., higher yield, thermal stability, flexibility in size, or so). It would be very promising to obtain reliable products through an accurate synthetic process. Herein, we present a comprehensive study for directly intermingling DNA blocks into higher DNA constructs through the 5′-end cohesive ligation of T4 ligase, a process called enzyme-guided DNA sewing. In particular, 5′ overhangs of DNA blocks were modulated in order to optimize their ligation efficiency by considering both thermodynamics and stereochemistry of four base pairs at the overhang site.

## Results

### Construction and characterization of DNA sewing material

**Figure 1:**
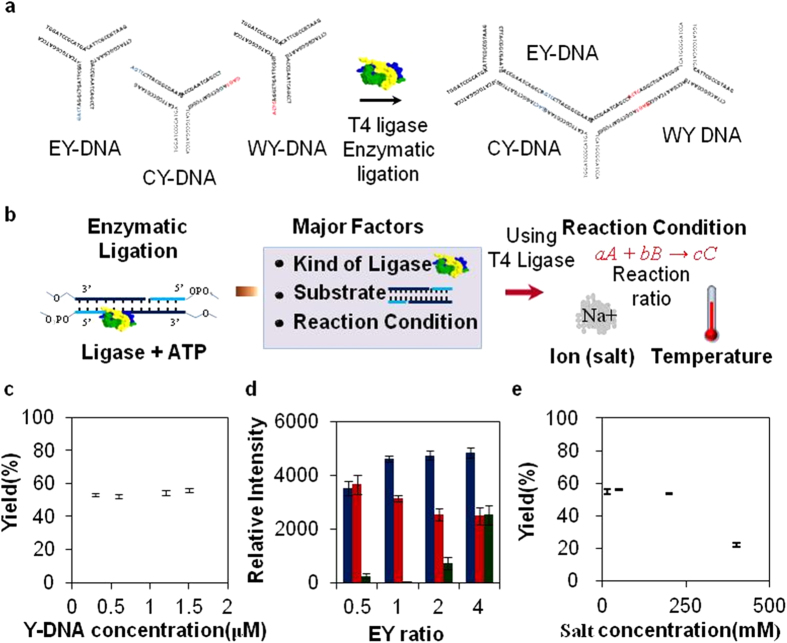
Schematic drawing of major ligation factors and evaluation of T4 ligase activity. (**a**) A schematic of DNA sewing material preparation. Each overhang sequence of WY-, EY- and CY-DNA blocks is ligated by T4 ligase. (**b**) Depiction of the ligation mechanism and three major ligation factors. These major factors were characterized by impact on ligation efficiency. (**c**) Various molar concentrations of Y-DNAs were tested with fixed amounts of adenosine triphosphate (1 mM) and T4 ligase (30 Weiss units). (**d**) Molar ratios of EY-DNA were changed under the fixed amount of WY-CY, which means the mixed solution of WY-DNA and CY-DNA in determined molar ratio. The concentration of WY-CY was fixed to 6 μM in ligation solution. The ratios of WY-CY to EY-DNA were 1:0.5, 1:1, 1:2 and 1:4 in sequence. Blue, red and green bars represent T-DNA, partial T-DNA and unreacted Y-DNA, respectively. (**e**) Various salt concentrations (15, 50, 100, 200 and 400 mM) were tested. Each data point represents the mean of triplicate experiments; error bars represent the SD.

Y-shaped DNA (Y-DNA) was used as a model DNA block for tree-shaped DNA assembly (T-DNA) as a simple DNA sewing material model (Fig. 1a and see Supplementary Fig. S1a,b online). More detailed sequence information is described in Table S1. Due to its intrinsic anisotropic property, T-DNA may possess two different Y-DNAs at two accessible positions of central Y-DNA (CY) in three different Y-DNAs. The Y-DNA at these different locations were designated as West Y-DNA (WY) and East Y-DNA (EY), respectively. To estimate the product efficiency of ligation, WY-CY and EY-CY were analyzed using a gel electrophoresis mobility shift assay (GEMSA) (see Supplementary Fig. S1c,d online).

To effectively synthesize the T-DNA model formed by the interlocking of three different Y-DNAs, several environmental parameters were first tested (Fig. 1b). Regarding the productivity of the ligase enzyme, a molar ratio of reactants and salt concentration were investigated. Various molar concentrations of Y-DNAs (0.3, 0.6, 1.2, and 1.5 μM) were successively tested with fixed amounts of adenosine triphosphate (1 mM) and T4 ligase (30 Weiss units) (Fig. 1c). However, no significant effects were observed in T-DNA formation. In addition, molar ratios of WY-CY to EY (1:0.5, 1:1, 1:2, and 1:4) were also varied under fixed amount of WY-CY (Fig. 1d). While only 0.3 μM of EY-DNA was used to make T-DNA with 0.6 μM of WY-CY, the band intensity of T-DNA should be much lower in the group of 0.5 ratios. Meanwhile, there were no significant differences observed among the groups of 1, 2 and 4 ratios. Several NaCl concentrations (15, 50, 100, 200 and 400 mM) were also tested at room temperature in order to determine the effect on product yield. This resulted in no deviations in T-DNA mass at concentrations lower than 200 mM. At higher NaCl concentrations, product yield decreased sharply, likely due to damaged T4 ligase (Fig. 1e)^16^,^17^.

The entire process of ligation is composed with protein-DNA recognition and catalytic reactions for nick sealing. In case of protein-DNA recognition process, the stereospecific interaction of protein on DNA substrates is a major concern. It is much known that the major and minor grooves in double stranded DNA structure play an important role in protein-DNA recognition^18^,^19^,^20^,^21^. There are two types of mechanism for recognizing specific DNA sequences by proteins: i) Hydrogen bonds with specific bases, and ii) Sequence-dependent deformations of the DNA helix. In addition, arginine residues of protein and minor grooves of DNA electrostatically bind to each other. Especially, its binding efficiency may be determined by the shape of minor groove in DNA structure. Likewise, DNA liagse and RNA ligase may be in contact with the minor groove in DNA helical structure. Additionally, a nick-joining in double stranded DNA by T4 DNA ligase involves three catalytic reaction steps: formation of enzyme-adenylate, formation of double stranded DNA-adenylate, and nich-sealing. It is inferred that the nick recognition and activity of DNA ligase at end points of two different overhangs may be dependent of both minor groove stereochemistry and thermodynamics^22^,^23^,^24^. Thus, characteristic thermodynamics and stereochemistry of DNA should be considered to identify new DNA sewing architectures through the enzymatic activity of ligase.

### End sequence-dependent investigation for the formation of DNA sewing nanostructure

In this study, the Gibbs energy and minor groove at end sequences were principally investigated with regard to sequence-dependent ligase efficiency. It is known that the width of minor groove varies depending on the sequence of nucleotides^20^. The distance between the phosphates backbones were critically affected by specific sequence arrangement with changes of negative electrostatic potentials along the minor grooves; AT-rich sequences tend to have more narrow minor grooves than GC-rich sequences. Likewise, Gibbs free energy, which indicates the thermodynamic states of DNA double helix structure, is also highly dependent on the sequence of DNA. Correspondingly, nearest neighbor thermodynamic parameters, which are described in the Supplementary Fig. S2 online, suggest that AT-rich sequences have less stable Gibbs free energy states than GC-rich sequences.

**Figure 2:**
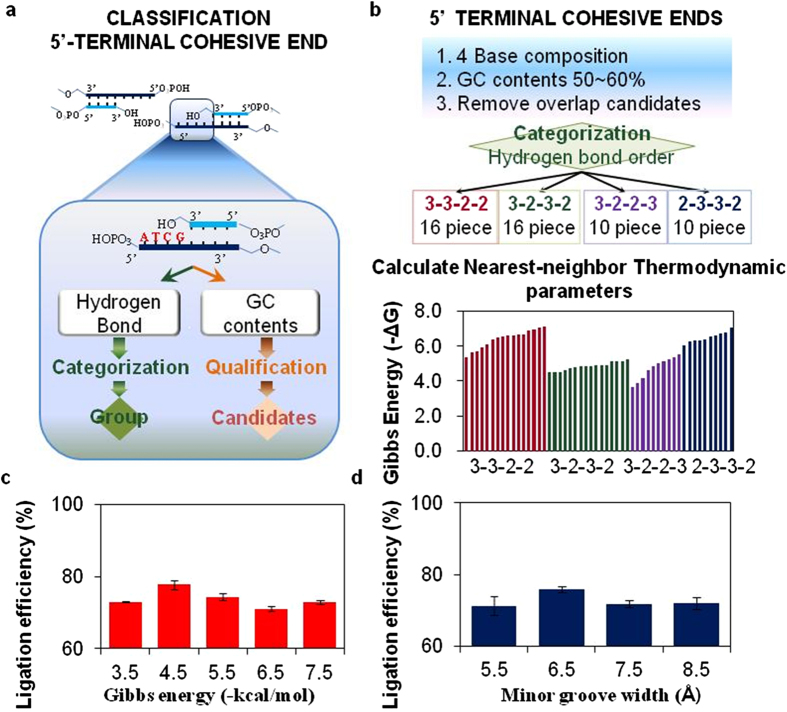
Ligation efficiency of 52 overhang sequences and their thermodynamic properties and helical structures. (**a**) Flow chart for overhang sequence classification. (**b**) Criteria for four-base overhang sequences to evaluate Gibbs energy. (**c**) Evaluation of ligation efficiency according to Gibbs energy (P = 0.0001). (**d**) Evaluation of ligation efficiency according to minor groove width (P = 0.0427). Data are represented as mean ± S.D.; ANOVA test.

Each overhang sequence in all candidates was selected to have 50% GC content, where it is expected to form a spiral helix^3^,^25^. Gibbs energies in all base-pair combinations were calculated using nearest neighbor base-base thermodynamics (see Supplementary Fig. S2 online)^26^,^27^,^28^,^29^. The base-pair combinations were classified into four subgroups according to hydrogen bonding order. It is known that the released Gibbs free energy of DNA is dependent on the sequential arrangement (Fig. 2a,b and see Supplementary Fig. S2 online). Fifty-two different complementary base pairs were introduced at the end sequence of a single Y-DNA in order to form T-DNAs. The ligation efficiencies were measured using estimated Gibbs energy. In addition, possible helix structures at minor grooves were predicted (see Supplementary Fig. S3 online). A significant increase in ligation efficiency was observed at specific energy values of −5.0 ~ − 4.0 kcal/mole and at minor groove widths of 6.0 ~ 7.0 Å (Fig. 2c,d). It is evident that the distinctive energy and minor groove structures of 5′-end cohesive base sequences are easily recognized by T4 ligase and activated to further seal nick sites.

Among the 52 pairs, only five different cohesive base pair sequences were tested and finally chosen for use in the association of thermodynamic properties of 5′-end cohesive base pairs sequences with T4 ligase efficiency (see Supplementray Fig. S4a online). These pairs were further tested to elucidate the effect of partial yield (exemplified as either WY-CY or EY-CY) and complete T-DNA. Among the pairs, the Gibbs energies of three pairs were equal or similar to each other. However, the remainder showed different Gibbs energies. T-DNA total yield is best produced around −4.52 kcal/mol (see Supplementary Fig. S4b online). It is strongly confirmed that the thermodynamic properties of DNA increase the efficiency of T4 ligase.

### Evaluation of abnormal sewing on DNA nanostructures

**Figure 3:**
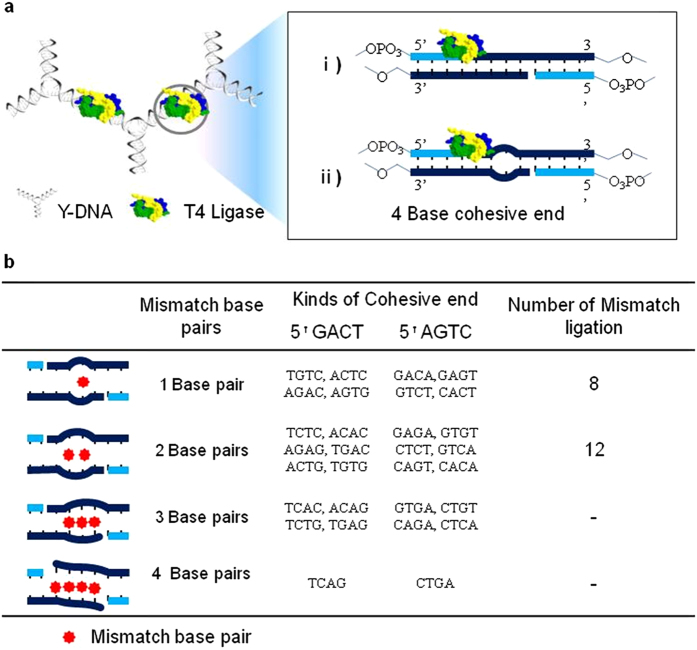
Profiling analysis of mismatch ligations. (**a**) Depiction of two different overhangs ligations. i) Normal ligation and ii) mismatch ligation with a non-complementary sequence pair at the cohesive end. (**b**) Possible mismatch ligations of GACT and its complementary sequence (AGTC). One to four mismatched base pairs were compared with several groups having 3232 hydrogen bond orders in which high ligation efficiency was expected. In the case of 1 and 2 mismatch base pairs, mismatch ligation was observed.

To further distinguish the differences in Gibbs energy of base sequences in the final product yield, mutual interactions of bases at end positions were profiled (Fig. 3a). Undesirable mismatches were intentionally positioned at the end sequences of Y-DNAs. Thermodynamic stability in unexpected base-base forms, which is comparable to that of a Watson-Crick, was observed^30^,^31^,^32^,^33^,^34^. Here, it is noted that two Y-DNA blocks possessing different overhang bases but the same body sequences are non-complementary to each other at either one or two bases or at a larger number of bases (Fig. 3b and see Supplementary Fig. S5 online). In cases of either one or two mismatched base pairs, partial T-DNAs were obtained in an unexpected manner. On the other hand, no mismatch ligation was observed when there were three or four mismatched bases pairs. With an increased number of mismatched bases pairs, the possibility of mismatch ligation dramatically decreased because of instability of thermodynamic property and helical structure.

**Figure 4:**
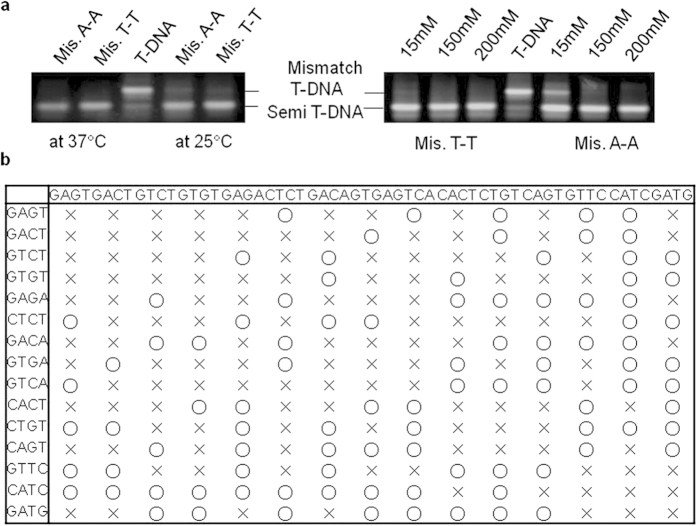
Optimization for the reduction of undesirable mismatch ligations. (**a**) GEMSA image of T-DNA and its mismatched compartments. Left experiment ligated under different temperature (25 °C and 37 °C) and right experiment ligated under different salt (15, 150 and 200 mM) (**b**) Guidelines for successful DNA nanostructures without adjustment of reaction environments (15 mM NaCl and 25 °C).

After investigation of abnormal ligation, we questioned why CY, which has two different cohesive ends of EY and WY, could induce EY-CY via mismatch ligation even though there is a WY binding site. It was significantly issued once we finely tune several ligation parameters in DNA assembly. Among the mismatch ligation cases (see Supplementary Fig. S5 online), AGTC and AGAC in each overhang of WY and EY were individually selected as the model sequences for the study of mismatch ligations. Undesirable ligations were also suspected to be caused by other environmental variables such as salt concentration and incubation temperature^11^,^32^,^33^,^34^. EY (AGAC) and CY (GACT and GTCT at both sites) were intentionally ligated with the expectation of the formation of partial T-DNA (CY-EY). A similar experiment was also carried out using WY (AGTC). However, a significant amount of complete T-DNA was observed, which may have been induced by the mismatch ligation of either GACT and AGAC or GTCT and AGTC. By adjusting the temperature and salt concentration, no such mismatch ligations were produced (Fig. 4a). It is noted that non-Watson-Crick base pairs were significantly decreased either at 37 °C or with salt concentrations above 150 mM. If a ligation is incubated at a higher temperature, it may result in cohesive base pair hybridization; such mismatch ligations are significantly suppressed. Moreover, it is assumed that salt affects the DNA helical structure, which may ultimately influence the activity of T4 ligase for recognition of certain sequences^32^,^34^. It may recover the final ligation efficiency, thus minimizing the mismatch ligation. In conclusion, some guidelines should be proposed for the construction of pure enzyme-guided DNA sewing nanostructures through the selective end cohesive ligation of DNA blocks (Fig. 4b). Irrespective of the environmental conditions during synthesis, a complete DNA construct was achieved through the simple interconnection of a few DNA blocks via ligase activity using the rules suggested in the table of Fig. 4; Gibbs free energy should be in the range of −4.0~ −5.0 kcal/mole in the four-base cohesive hybridization, followed by no mismatched base pairs. These rules can be applied to the creation of a networked DNA sewing nanoconstruct based on any type of end cohesive sequence.

### Identification of *in vivo* cytosolic RNA markers with functional DNA sewing nanomaterials

**Figure 5:**
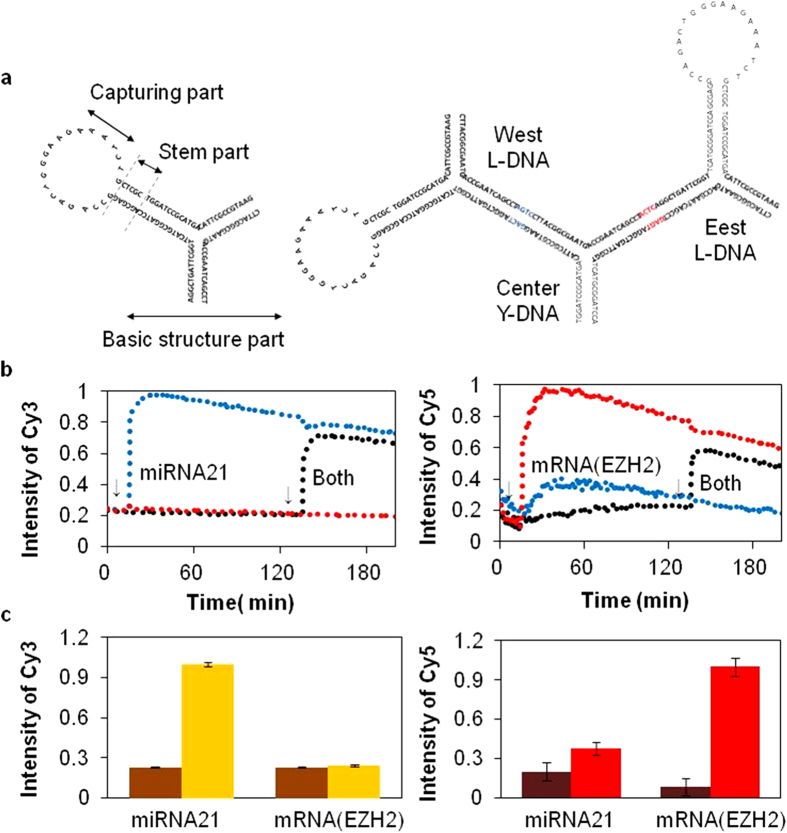
Description and fluorescence measurements of functional DNA sewing materials. (**a**) Schematic drawings of two different functional DNA sewing materials such as L-DNA and LT-DNA. (**b**) Functional time-dependent assay for LT-DNA. Two graphs show the fluorescence intensity of LT-DNA. Left and right graphs represent Cy3 and Cy5, respectively. Blue line indicates LT-DNA with the complementary sequence miRNA21 added at 30 min. Red line indicates LT-DNA with the complementary sequence mRNA (EZH2) added at 30 min, and black line indicates LT-DNA with both complementary sequences added after 2.5 hour. The intensities of Cy3 and Cy5 increased when the complementary sequence was mixed at certain times (arrows) in the multi-LT-DNA solution (30 min and 2.5 hour). (**c**) Maximum fluorescence values of Cy5 and Cy5 were compared. Each data point represents the mean of triplicate experiments; error bars represent the SD.

To practically evaluate the complete T-DNAs suggested, we used them as novel diagnostic probes. Some functional modules containing a loop structure with 31 bases were appended (Table S2a) to specifically identify cancer-specific RNA markers. Such functional Y-DNAs were abbreviated as L-DNA and were ligated to the central Y-DNA for formation of functional T-DNA (Fig. 5a), represented as LT-DNA. This formation was confirmed using GEMSA and was compared to normal T-DNAs (see Supplementary Fig. S6 online). Upon the addition of a single oligonucleotide complementary to the loop sequence of L-DNA, a gel band was produced, indicating that the supplementary single nucleotides were successfully captured by the loop sequence of LT-DNA. Inspired by these achievements, two different L-DNAs corresponding to EZH2 encoding messenger RNA and microRNA 21 (termed miRNA21) were tested as specific breast cancer markers. These sequences were simultaneously ligated to either side of a CY to form a versatile LT-DNA (Table S2b indicates the sequence information of all oligonucleotides used in the L-DNAs). The LT-DNAs were shown to have potential to selectively capture target oligo-ligands.

In addition to the stem-loop structure, L-DNAs containing either Cy3^TM^ or Cy5^TM^ fluorescent dyes were created. The fluorescence signal was quenched by working solutions such as Iowa Black^®^ RQ through fluorescence resonance energy transfer (FRET) (Fig. 5b,c). When such RNA markers react with the stem-loop structure of LT-DNA, the configuration opens into a linear shape, producing very strong fluorescence emissions that can be observed via FRET; the emission strengths of Cy3^TM^ and Cy5^TM^ were amplified by five- and two-fold, respectively. More interestingly, two different RNA markers corresponding to EZH2 and miR21 were simultaneously detected using the LT-DNAs; addition of the EZH2 RNA marker induced no Cy3^TM^ enhancement, while miR21 did not show fluorescent emissions in Cy5^TM^. These results strongly indicate that this versatile DNA nanoconstruct can be used for multi-detection of several RNA markers with high selectivity and sensitivity.

## Discussion

In this study, we proposed predictive criteria for optimized selection of 5′-end overhang sequences for the directed assembly of DNA blocks through enzymatic ligation. Through the consideration of thermodynamics and stereochemistry on 5′ overhang sequences, the yield and purity were significantly influenced. In addition, this data provides evidence on the essential role of DNA substrates in DNA-T4 ligase recognition and its activation mechanism. Using fluorescence codes, we investigated the anisotropicity and cancer-diagnostic capacity of the DNA constructs. Such a predictive model will allow the design of new interweaved DNA materials for nucleic acid-based bioapplications including genetics and gene sequencing.

## Materials and Methods

### Synthesis of model DNA sewing materials

To synthesize a Y-DNA block, three different oligonucleotides were designed and manufactured. Table S1 demonstrates the T-DNA and Y-DNA sequence information. All oligonucleotides were provided by Integrated DNA Technologies (IDT, Inc., Coralville, IA). Y-DNA (6 μM) was annealed in a buffer composed of 50 mM NaCl, 10 mM Tris-HCl (pH 8.0) and 0.1 mM EDTA. To construct T-DNA, sequences were formed via a complementary hybridization of each base, followed by T4 ligase (Promega, Madison, WI). To synthesize T-DNA, three Y-DNAs possessing 5′-cohesive ends were ligated with T4 ligases in 10× ligase buffer. Specifically, 0.6 μM Y-DNAs were combined with T4 ligase (2 μl) and 10× ligase buffer (5 μl) in a total volume of 50 μl and reacted for 3 hours at 25 °C, overnight at 4 °C or for 1 hour at 37 °C as suggested in a user’s manual.

### Imaging analysis

The DNA products were evaluated by analyzing gel electrophoretic images of 3% agarose gel. Gel electrophoresis was performed under 100 V for 40 min, and the gels were immediately stained with ethidium bromide (EtBr) (2 μg/ml) for 20 min. The gel images were visualized using a GELDoc-it imaging system under Launch VisionWorksLS UPV and were analyzed using TotalLab Quant gel quantification software version 2.01, provided by ImageMaster (TotalLab Ltd., Newcastle upon Tyne, UK). Yields and ligation efficiency were compared as product bands divided by total bands. T-DNAs moved more through the gels compared with Y-DNAs, indicating the higher molecular weight of T-DNA (79713.2 Da) compared to Y-DNA (26902.5 Da).

### Optimization parameters in DNA sewing

To examine the effect of environment, ligation conditions including Y-DNA reactants versus adenosine triphosphate, reactant ratio and salt concentration were varied. The effect of DNA concentration with fixed amounts of adenosine triphosphate and enzyme was examined. Different amounts of Y-DNAs (0.3 μM, 0.6 μM, 1.2 μM and 1.5 μM) were combined with T4 ligase (2 μl) and 10× ligase buffer(5 μl) in a total volume of 50 μl. Next, the reactant ratio of CY: WY: EY was changed from 1:1:0.5 to 1:1:1, 1:1:2 and 1:1:4. In this experiment, ligation efficiency was difficult to measure since the remaining Y-DNA induced error. Instead, the intensity of the DNA bands was measured. Salt concentration was tested at 15, 50, 100, 200 and 400 mM.

### Calculation of Gibbs energy of 5′ cohesive bases

Gibbs energy of cohesive ends was calculated with nearest neighbor thermodynamic properties. Gibbs energies were further verified using the IDT internet service ‘oligo analyzer 3.1 tool.’ Gibbs energies were measured at 6 μM Oligo, 15 mM Na^+^, 0 mM Mg^2+^, 0 mM dNTPs concentrations.

### Design of functional DNA sewing materials and evaluation of their target binding ability

New stem-loop DNA was designed such that its characteristic functionality was supplemented into Y-DNA. One oligonucleotide contained an additional loop with 31 bases, which was capable of specifically identifying cancer-specific RNA markers depending on the sequence. L-DNA consisted of three parts: (i) basic structural part of Y-DNA, (ii) capturing part that binds target DNA and (iii) stem part (five base pairs) that maintains the loop structure. Using two high ligation efficiency overhang sequences (GACT and GAGT), LT-DNA was synthesized. The synthesis protocol is the same as that of T-DNA (L-DNAs in 0.6 μM were combined with with T4 ligase (2 μl) and 10× ligase buffer (5 μl) in a total volume of 50 μl at 37 °C. After the construction of LT-DNA, target DNA (6 × 10^−5^ μmol) complementary to the capturing part sequences was added to the LT-DNA solution at 37 °C. LT-DNA captured target DNAs for four hours. After the reaction, products were evaluated using GEMSA.

### Fluorescence measurements of multifunctional DNA materials

Two oligonucleotides with a modified 5′ end with dark quencher (Iowa black RQ) and Cyanine dyes (Cy3 or Cy5) were provided by Integrated DNA Technologies (IDT, Inc., Coralville, IA) (Table S2b). After the loop-stem structure was formed, Cy3 or Cy5 reacted with quencher (Iowa Black RQ) to mute the fluorescence intensity of the cyanine dyes. After target RNAs were probed by LT-DNA, the loop structures were disrupted, and the fluorescence increased. All measurements were made in 100 μl solutions containing 0.6 μM LT-DNA with twice (12 × 10 μmol) the molar amount of complementary target RNA (mRNA EZH2 and miRNA21) from its LT-DNA. The fluorescence of these reaction mixtures was measured by excitation with a 512 nm and 550 nm laser light source in a SpectraMax M5 (Molecular Devices, Sunnyvale, CA). Cy3 was measured with excitation at 512 nm/emission at 614 nm, and Cy5 was measured with excitation at 650/emission at 670 nm. The temperature was fixed at 37 °C, and each reaction lasted for four hours. Fluorescence was measured every 30 sec during the reaction.

## Additional Information

**How to cite this article**: Song, I. H. *et al.* Enzyme-guided DNA Sewing Architecture. *Sci. Rep.* **5**, 17722; doi: 10.1038/srep17722 (2015).

## Supplementary Material

Supplementary Information

## References

[b1] Seeman N. C. De novo design of sequences for nucleic acid structural engineering. J Biomol Struct Dyn 8, 573–581 (1990). 2100519 10.1080/07391102.1990.10507829

[b2] Seeman N. C. DNA Nanotechnology: Novel DNA constructions. Annu Rev Biophys Biomol Struct 27, 225–248 (1998). 9646868 10.1146/annurev.biophys.27.1.225

[b3] Luo D. The road from biology to materials. Materials Today 6, 38–43 (2003).

[b4] Goodman R. P. *et al.* Reconfigurable, braced, three-dimensional DNA nanostructures. Nat Nanotechnol 3, 93–96 (2008). 18654468 10.1038/nnano.2008.3

[b5] Castro C. E. *et al.* A primer to scaffolded DNA origami. Nat Methods 8, 221–229 (2011). 21358626 10.1038/nmeth.1570

[b6] Douglas S. M. *et al.* Self-assembly of DNA into nanoscale three-dimensional shapes. Nature 459, 414–418 (2009). 19458720 10.1038/nature08016 PMC2688462

[b7] Wang Z. G. & Ding B. DNA-based self-assembly for functional nanomaterials. Adv Mater 25, 3905–3914 (2013). 24048977 10.1002/adma.201301450

[b8] Song J. *et al.* Direct visualization of transient thermal response of a DNA origami. J Am Chem Soc 134, 9844–9847 (2012). 22646845 10.1021/ja3017939

[b9] Hahn J. Wickham S. F. J., Shih W. M. & Perrault S. D. Addressing the instability of DNA nanostructures in tissue culture. ACS Nano 8, 8765–8775 (2014). 10.1021/nn503513p PMC4174095 25136758

[b10] Kim H. *et al.* Stability of DNA Origami Nanostructure under Diverse Chemical Environments. Chem. Mater. 26, 5265–5273 (2014).

[b11] Marchi A. N. *et al.* Toward Larger DNA Origami. Nano lett. 14, 5740–5747 (2014). 25179827 10.1021/nl502626s

[b12] Rajendran A. *et al.* Photo-cross-linking- assisted thermal stability of DNA origami structures and its application for higher-temperature self-assembly. J. Am. Chem. Soc. 133, 14488–14491 (2011). 21859143 10.1021/ja204546h

[b13] O’Neill P., Rothemund P. W. K., Kumar A. & Fygenson D. K. Sturdier DNA nanotubes via ligation. Nano lett. 6, 1379–1383 (2006) 10.1021/nl0603505 16834415

[b14] Ackermann D. *et al.* A double-stranded DNA rotaxane. Nat Nanotechnol 5, 436–442 (2010). 20400967 10.1038/nnano.2010.65

[b15] LaBean T. H. *et al.* Construction, analysis, ligation, and self-assembly of DNA triple crossover complexes. J Am Chem Soc 122, 1848–1860 (2000).

[b16] Sambrook J., Fritsch E. F. & Maniatis T. Molecular cloning: a laboratory manual, 2nd. eds Lanssen K. A. (Cold Spring Harbor Laboratory Press, New York) (1989).

[b17] Landegren U., Kaiser R., Sanders J. & Hood L. A. A ligase-mediated gene detection technique. Science 241, 1077–1080 (1988). 3413476 10.1126/science.3413476

[b18] Seeman N. C., Rosenberg J. M. & Rich A. Sequence-specific recognition of double helical nucleic acids by proteins. Proc Natl Acad Sci USA 73, 804–808 (1976). 1062791 10.1073/pnas.73.3.804 PMC336007

[b19] Garvie C. W. & Wolberger C. Recognition of specific DNA sequences. Mol Cell 8, 937–946 (2001). 11741530 10.1016/s1097-2765(01)00392-6

[b20] Rohs R. *et al.* The role of DNA shape in protein-DNA recognition. Nature 461, 1248–1253 (2009). 19865164 10.1038/nature08473 PMC2793086

[b21] Tullius T. Structural biology: DNA binding shapes up. Nature 461, 1225–1226 (2009). 19865161 10.1038/4611225a

[b22] Cherepanov A. V. & de Vries S. Kinetics and thermodynamics of nick sealing by T4 DNA ligase. Eur J Biochem 270 4315–4325 (2003). 14622296 10.1046/j.1432-1033.2003.03824.x

[b23] Cherepanov A. V. & de Vries S. Dynamic mechanism of nick recognition by DNA ligase. Eur J Biochem 269 5993–5999 (2002). 12473094 10.1046/j.1432-1033.2002.03309.x

[b24] Cotner-Gohara E., Kim I. K., Tomkinson A. E. & Ellenberger T. Two DNA-binding and nick recognition modules in human DNA ligase III. J Biol Chem 283, 10764–10772 (2008). 18238776 10.1074/jbc.M708175200 PMC2447648

[b25] Luo D., Cu Y. T., Li Y. & Um S. H. DNA Vaccines : Methods and protocols, eds W. M. Sltzman, H. Shen & J. L. Brandsma (Humana Press, New Jersey), Chapter 10 (2006).

[b26] Yakovchuk P., Protozanova E. & Frank-Kamenetskii M. D. Base-stacking and base-pairing contributions into thermal stability of the DNA double helix. Nucleic Acids Res 34, 564–574 (2006). 16449200 10.1093/nar/gkj454 PMC1360284

[b27] SantaLucia J. & Hicks D. The thermodynamics of DNA structural motifs. Annu Rev Biophys Biomol Struct 33, 415–440 (2004). 15139820 10.1146/annurev.biophys.32.110601.141800

[b28] Bommarito S., Peyret N. & SantaLucia J. Thermodynamic parameters for DNA sequences with dangling ends. Nucleic Acids Res 28, 1929–1934 (2000). 10756193 10.1093/nar/28.9.1929 PMC103285

[b29] Breslauer K. J., Frank R., Blockerm H. & Marky L. A. Predicting DNA duplex stability from the base sequence. Proc Natl Acad Sci USA 83, 3746–3750 (1986). 3459152 10.1073/pnas.83.11.3746 PMC323600

[b30] Werntges H., Steger G., Tiesner D. & Fritz H. J. Mismatches in DNA double strands: thermodynamic parameters and their correlation to repair efficiencies. Nucleic Acids Res 14, 3773–3790 (1986). 3714496 10.1093/nar/14.9.3773 PMC339814

[b31] Aboul-ela F., Koh D., Tinoco I. & Martin F. H. Base-base mismatches. Thermodynamics of double helix formation for dCA3XA3G + dCT3YT3G(X, Y = A,C,G,T). Nucleic Acids Res 13, 4811–4824 (1985). 4022774 10.1093/nar/13.13.4811 PMC321828

[b32] Kim J. H. & Mrksich M. Profiling the selectivity of DNA ligases in an array format with mass spectrometry. Nucleic Acids Res 38, e2 (2009). 19854942 10.1093/nar/gkp827 PMC2800213

[b33] Wu D. Y. & Wallace R. B. Specificity of the nick-closing activity of bacteriophage T4 DNA ligase. Gene 76, 245–254 (1989). 2753355 10.1016/0378-1119(89)90165-0

[b34] Luo J., Bergstrom D. E. & Barany F. Improving the fidelity of Thermus thermophilus DNA ligase. Nucleic Acids Res 24, 3071–3078 (1996). 8760896 10.1093/nar/24.15.3071 PMC146030

