# Supplementary material for: Enzyme-guided DNA Sewing Architecture
Source: Sci Rep. 2015 Dec 4;5:17722. doi: 10.1038/srep17722 (PMC4669507; doi:10.1038/srep17722)
Supplement: Supplementary Information [file srep17722-s1.doc]

Supplementary Information for

Enzyme-guided DNA Sewing Architecture

In Hyun Song, Seung Won Shin, Kyung Soo Park, Yves Lansac, Yun Hee Jang &

Soong Ho Um*

Mr. I.H. Song, Ms. S.W. Shin, K.S. Park,

School of Chemical Engineering

Sungkyunkwan University

Suwon, Gyeonggi-do, 440746, South Korea

Prof. S.H. Um

SKKU Advanced Institute of Nanotechnology (SAINT)

School of Chemical Engineering

Sungkyunkwan University

Suwon, Gyeonggi-do, 440746, South Korea

E-mail: [sh.um@skku.edu](mailto:sh.um@skku.edu)

Prof. Y. Lansac.
Laboratoire d’Electrodynamique des Matériaux Avancés

Université François Rabelais

Parc Grandmont Tours, 37200, France

Prof. Y.H. Jang

School of Materials Science and Engineering

Gwangju Institute of Science and Technology

Gwangju, 500712, South Korea

**TABLE OF CONTENTS**

Supplementary Information Figures and Tables

Figure S1 Ligation efficiencies of T-DNA and its components

Figure S2 Rules for 5’ overhang sequences and calculation of Gibbs energy.

Figure S3 Ligation efficiency of 52 overhang sequences and their thermodynamic properties and helical structures.

Figure S4 Gibbs energy and ligation efficiency of partial and complete T-DNA.

Figure S5 Profiling analysis of mismatch ligations

Figure S6 Formation of Loop T-DNA

Table S1 Preparation of oligonucleotides for T-DNA blocks

Table S2 Preparation of oligonucleotides for LT-DNA blocks

Supplementary Infomation References

**Figure S1.** **Ligation efficiencies of T-DNA and its components.**


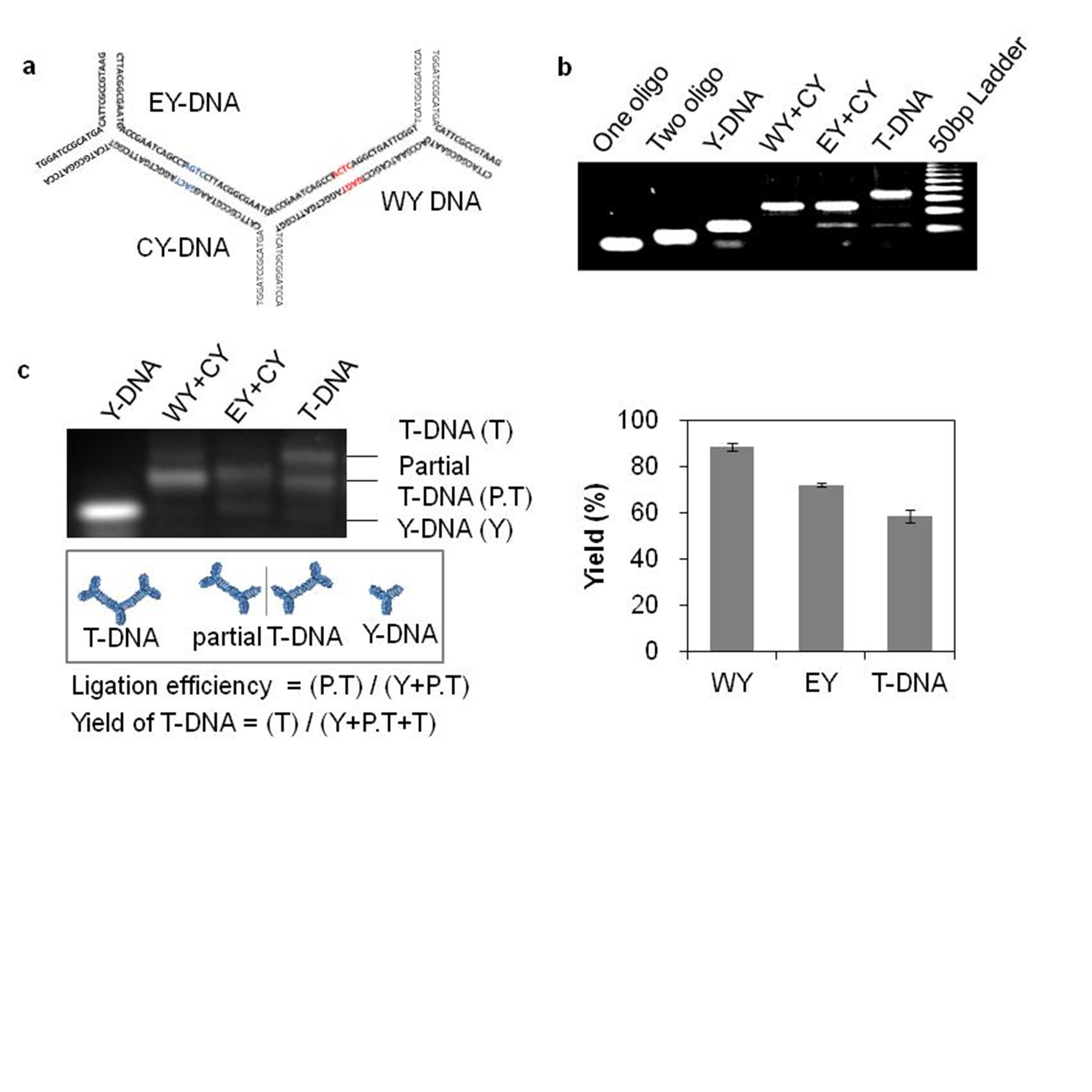


(a) A schematic drawing of T-DNA, which is comprised of EY-, WY- and CY-DNA. Two different overhang sequences were ligated for the formation of T-DNA. (b) Changes in gel electrophoretic mobility of T-DNA components. Each component showed stepwise increases in mobility with assembly into higher structures. (c) Ligation yield analysis. Gel electrophoretic image represents T-DNA and partial components. Each product yield was evaluated by comparing the relative band intensities. Calculated results are shown in the bar graph.

**Figure S2. Rules for 5’ overhang sequences and calculation of Gibbs energy.** In order to maintain the spiral structure of Y-shaped DNAs, their body sequences were designed to contain a GC content around 50 to 60%. This confines helical nanostructures of Y-DNA into B-type spiral structures rather than A- or Z-type spiral structures^1-3^. Thus, overhang sequences having 50% GC content were selected as candidates in this study. Consequently, 52 overhang sequences were ultimately chosen for this experiment (Supplementary Figure S4) and were classified according to sequence arrangements of hydrogen bonds between complementary nucleotides because the nearest neighbor thermodynamic properties are affected by successive location of purines and pyrimidines^4-6^. After confirming all experimental candidates, the Gibbs energy of each overhang sequence was calculated with consideration of nearest neighbor thermodynamic properties as suggested in the following equation:

ΔG_Total_ = - (Δg_i_+Δg_sym_)+Σ_x_ΔG˚

(Δg_i_ : helix initiation free energy, Δg_sym_ : free energy of self-complementary sequence or two complementary sequences, ΔG˚ : nearest neighbor thermodynamic properties). A method for calculation of Gibbs free energy is represented. Each theoretically calculated Gibbs free energy was verified using Integrated DNA Technologies (IDT) internet service ‘oligo analyzer 3.1 tool.’


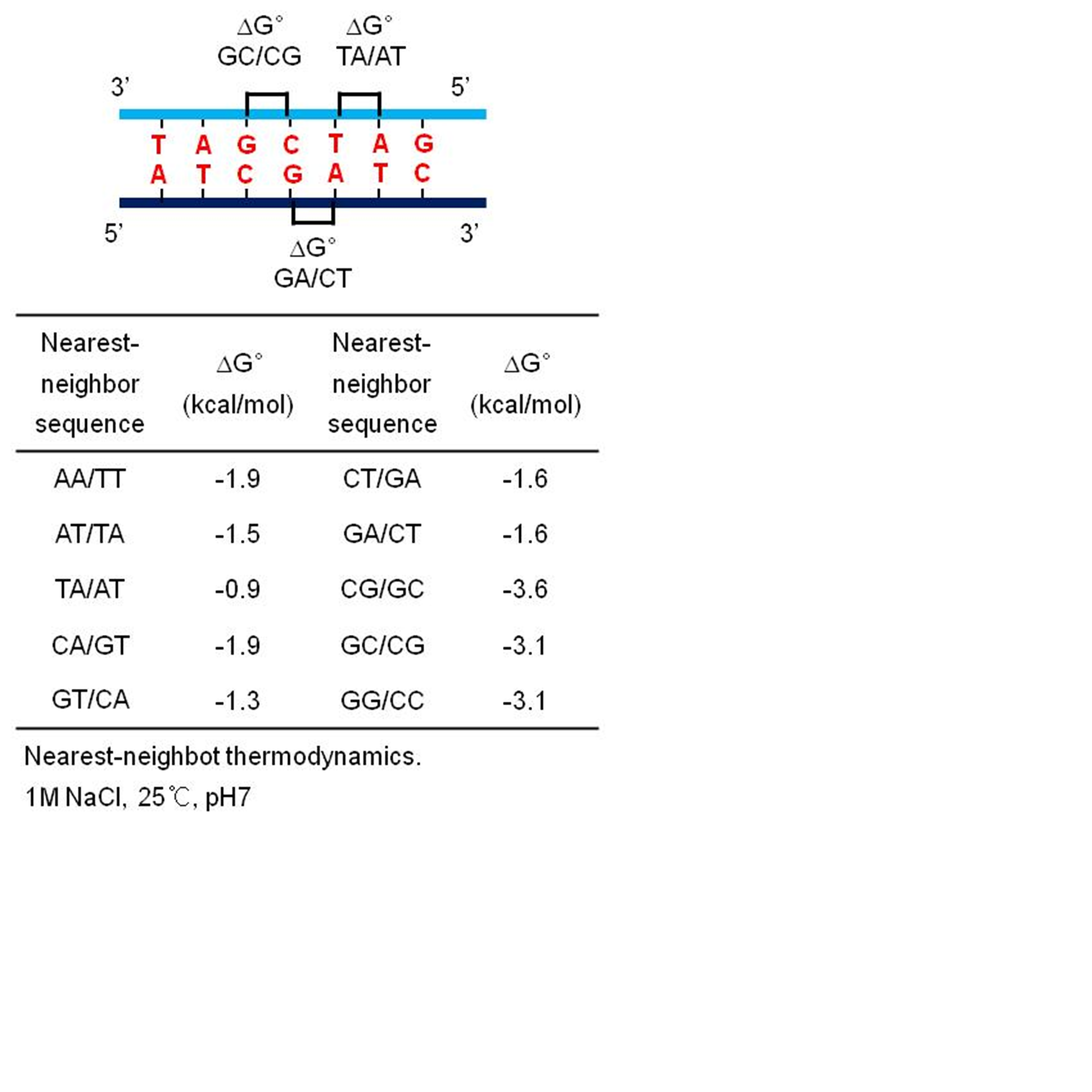


**Figure S3.** **Ligation efficiency of 52 overhang sequences and their thermodynamic properties and helical structures.** Based on a study conducted by Barry Honig 52 different DNA helical structures could be created at the overhangs, containing a minor groove of four base pairs rearranged in order to compare the relationships between T4 ligase enzyme activity and DNA shape^7^. Quoted values of minor groove width, which is the length of the minor groove containing bases that interact with T4 ligase, may not accurately reflect the actual minor groove width containing the cohesive end since the minor groove width of a DNA-protein complex has different helical structures from the actual overhang sequence. However, it was strongly suggested that the specific states of the DNA substrate enhanced the recognition and activation of T4 ligase. As a result, overhang sequences, which have their own Gibbs free energy between -5.0 kcal/mole and -4.0 kcal/mole, showed much higher ligation efficiencies compared with non-overhang sequences. Also, the peak point is located nearby -4.52 kcal/mole of Gibbs energy. Overhang sequences having a minor groove width between 6.0 Å ~ 7.0 Å showed better optimized efficiencies, but they did not have a significant impact as compared with that of ligation Gibbs energy. Each data point represents the mean of triplicate experiments; error bars represent the SD.

| **Group** | **Sequence** | **Ligation efficiency (%)** | **ΔG (kcal/mol)** | **Minor groove width(Å)** | **Sequence** | **Ligation efficiency (%)** | **ΔG (kcal/mol)** | **Minor groove width(Å)** |
| --- | --- | --- | --- | --- | --- | --- | --- | --- |
| **3322** | GGTA | 78.23±0.6 | -5.37 | 6.7 | GGAA | 69.88±0.7 | -6.59 | 6.8 |
|  | CCTA | 69.17±1.5 | -5.63 | 7.1 | CCTT | 72.38±1.4 | -6.61 | 6.7 |
|  | GCTA | 73.33±0.2 | -5.70 | 6.8 | CGAT | 74.01±1.7 | -6.66 | 6.8 |
|  | CGTA | 73.63±3.7 | -5.91 | 8.9 | GCTT | 74.06±2.3 | -6.68 | 7.5 |
|  | GGAT | 63.33±2.2 | -6.12 | 5.8 | CGTT | 73.24±0.3 | -6.90 | 5.6 |
|  | GGTT | 67.07±2.8 | -6.36 | 6.5 | CCAA | 69.61±1.4 | -6.97 | 7.7 |
|  | CCAT | 71.56±1.4 | -6.50 | 7.5 | GCAA | 72.05±1.0 | -7.04 | 7.6 |
|  | GCAT | 72.21±0.8 | -6.57 | 6.5 | CGAA | 73.86±1.8 | -7.13 | 6.9 |
| **3232** | GACT | 84.67±0.3 | -4.52 | 6.5 | GTGA | 76.45±1.6 | -4.87 | 6.1 |
|  | GAGT | 86.89±2.4 | -4.52 | 6.4 | CACT | 76.46±1.0 | -4.89 | 7 |
|  | GTCT | 84.42±2.0 | -4.52 | 6.1 | CAGT | 75.18±4.1 | -4.89 | 6.5 |
|  | GTGT | 81.99±0.1 | -4.64 | 6.9 | CTGT | 78.74±0.1 | -4.89 | 6.9 |
|  | GAGA | 74.91±0.5 | -4.75 | 6.2 | CAGA | 77.37±2.7 | -5.13 | 6.2 |
|  | CTCT | 79.62±1.6 | -4.77 | 6.8 | CTCA | 74.19±2.4 | -5.13 | 5.7 |
|  | GACA | 79.96±2.4 | -4.87 | 6.6 | CTGA | 77.71±1.1 | -5.13 | 7.5 |
|  | GTCA | 76.55±1.0 | -4.87 | 7 | CACA | 79.97±3.5 | -5.25 | 7 |
| **3223** | GTAC | 77.37±4.2 | -3.65 | 6.2 | GATG | 72.55±1.7 | -5.00 | 6.1 |
|  | GTAG | 72.80±1.2 | -3.90 | 7.3 | GAAG | 74.50±4.0 | -5.12 | 6.4 |
|  | CTAG | 72.61±1.0 | -4.16 | 6.5 | CAAC | 73.38±2.8 | -5.24 | 6.9 |
|  | GATC | 81.16±3.4 | -4.62 | 6.1 | CATG | 72.47±2.4 | -5.38 | 6 |
|  | GAAC | 74.25±1.5 | -4.86 | 5.5 | CAAG | 75.01±2.3 | -5.50 | 6.5 |
| **2332** | AGGT | 73.08±0.6 | -6.01 | 6.4 | ACGA | 66.83±3.3 | -6.53 | 7.5 |
|  | AGGA | 71.80±4.4 | -6.24 | 6.5 | TGGA | 67.31±3.4 | -6.60 | 7.2 |
|  | ACGT | 72.89±4.3 | -6.30 | 7 | AGCA | 72.68±0.4 | -6.69 | 7.9 |
|  | AGCT | 75.02±4.5 | -6.34 | 7.5 | TCGA | 70.21±3.1 | -6.76 | 8.4 |
|  | ACCA | 71.45±3.5 | -6.37 | 7.1 | TGCA | 72.65±5.1 | -7.05 | 7.5 |

**
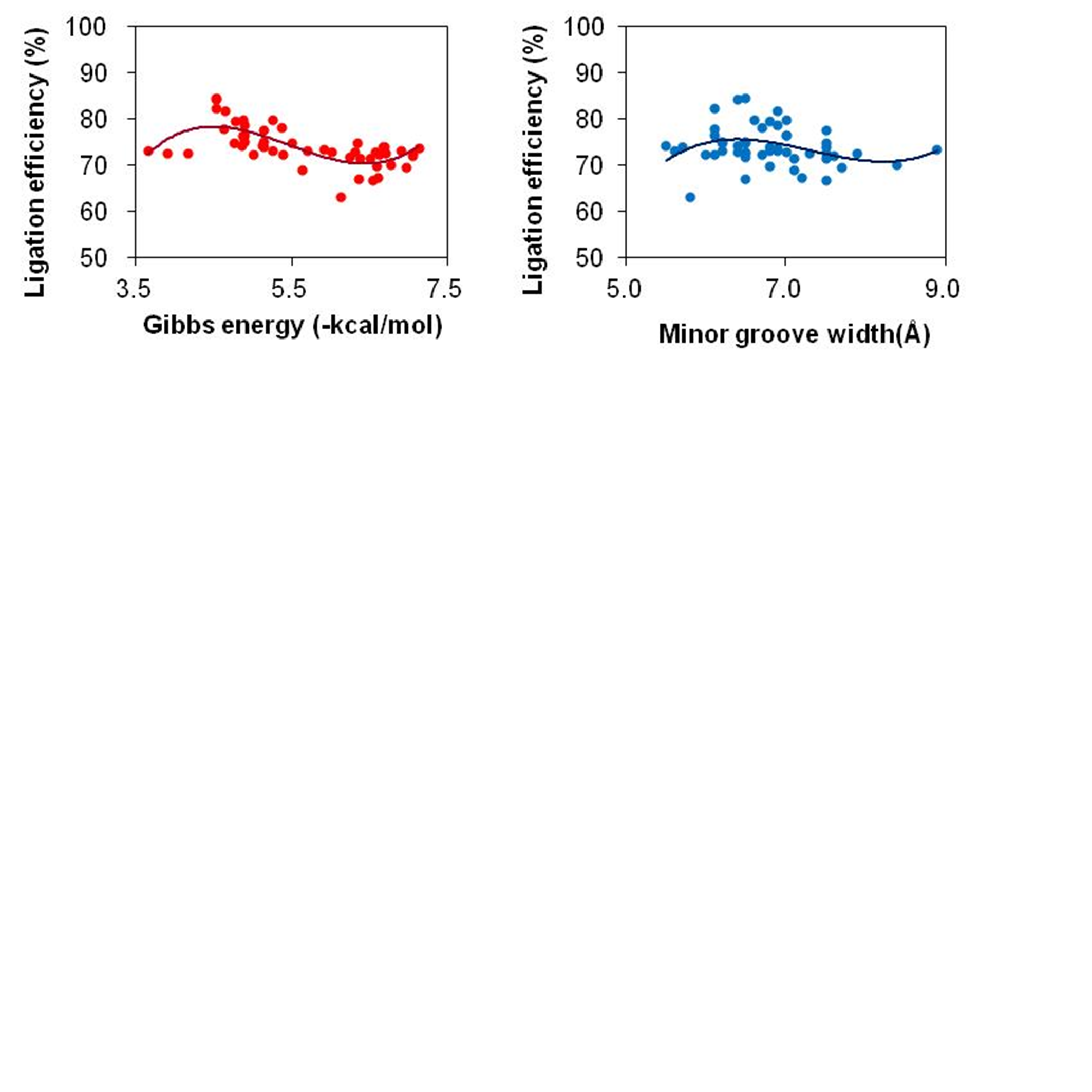
**

**Figure S4.** **Gibbs energy and ligation efficiency of partial and complete T-DNA.** To confirm the relationship between thermodynamic properties and pure yield of T-DNA, we selected a few candidates among the 52 overhang sequences. The overhang sequence (GACT) of WY showed substantial ligation efficiency for the formation of partial T-DNA (WY-CY) and was selected as a control sequence. Regarding the overhang sequences of EY, five base sequences were chosen. (a) Flow diagram for the selection of five overhang sequences for EY. Three candidates (GAGT, GTCT, and GATC) were selected as similar Gibbs energies with GACT of WY with following descriptions. 1) Gibbs energy range: To find another proper cohesive ends at the highest yield, Gibbs energy range was selected by - 4.0 to - 5.0 kcal/mole. 2) Removal of repetitive sequences: Repetitive base sequences such as GAGA was removed as shown in the Fig. S4. Even though, the repetitive base sequences were considered to have no great influences on total yields of ligation, considering the fact that the repetitive sequences could have a probable undesirable state. 3) Successive three base pairs must be different from bases of GACT in WY: To reduce some error probabilities, three successive bases should not be the same in WY-DNA as the candidates are selected. After three above criteria, all candidates with either similar or same Gibbs energy were selected. In order to effectually compare the relationship between Gibbs energy and ligation efficiency, maximal and minimal Gibbs energy candidates having similar minor groove width of new three base candidates were selected in other groups of others. (b) GEMSA of seven additional overhang sequences was conducted to calculate the ligation efficiency. In the case of T-DNA, the overhang sequence of WY was fixed, while that of EY was changed. Four different overhang sequences for EY were compared.


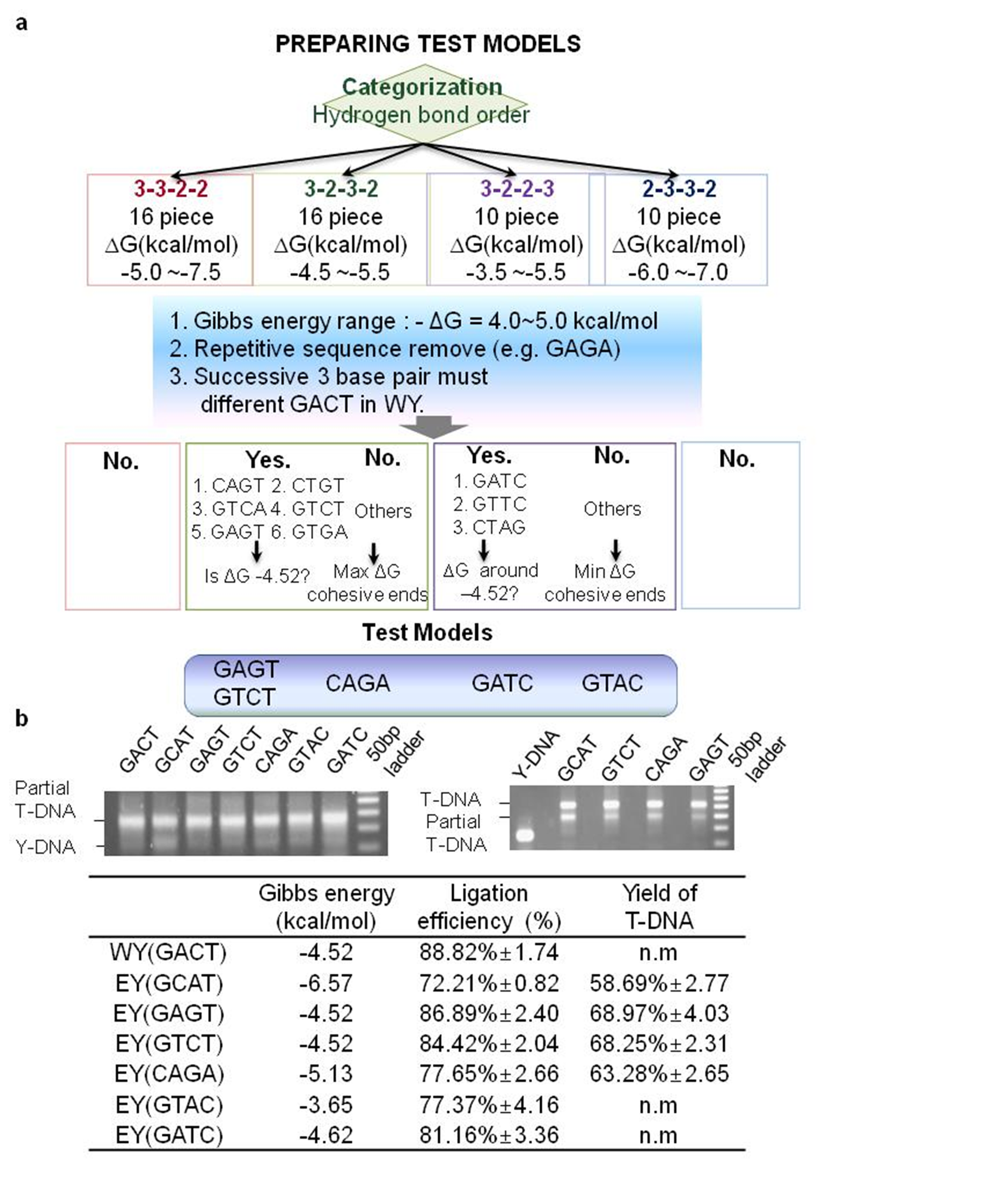


**Figure S5. Profiling analysis of mismatch ligations. Possible mismatch ligations of GACT (note that its complementary sequence is AGTC), which is located at the WY binding site. Other non-complementary sequences were tested. One to four mismatched base pairs were compared with several groups having 3232 hydrogen bond orders in which high ligation efficiency was expected. GEMSA image of mismatch ligations is shown, and their ligation efficiency results are presented in the table. Red letters indicate a mismatched base in the complementary sequence. Each data point represents the mean of triplicate experiments; error bars represent the SD.**

| **Number of mismatch base pairs** | **Sequence** | **Complementary sequence** | **Kind of mismatch base pairs** | **Ligation efficiency at 4**°C **(%)** | **Ligation efficiency at 25**°C **(%)** |
| --- | --- | --- | --- | --- | --- |
| **1 Base pair** | 5’ AGTC | 5’ GACA | A-A | 51.26±0.3 | 40.10±0.5 |
|  |  | 5’ GAGT | G-G | 62.34±0.5 | 51.13±0.1 |
|  |  | 5’ GTCT | T-T | 61.28±0.3 | 53.10±0.1 |
|  |  | 5’ CACT | C-C | 60.07±0.3 | 45.00±0.2 |
|  | 5’ GACT | 5’ TGTC | T-T | 64.95±0.7 | 63.95±1.0 |
|  |  | 5’ ACTC | C-C | 41.13±0.8 | 22.20±0.1 |
|  |  | 5’ AGAC | A-A | 60.83±1.4 | 55.09±0.7 |
|  |  | 5’ AGTG | G-G | 58.21±1.1 | 53.20±0.5 |
| **2 Base pairs** | 5’ AGTC | 5’ GAGA | G-G / A-A | 15.47±0.3 | 16.92±0.6 |
|  |  | 5’ GTGT | T-T / G-G | 11.67±1.1 | 17.71±0.4 |
|  |  | 5’ CTCT | C-C / T-T | 14.02±0.9 | 24.99±0.8 |
|  |  | 5’ GTCA | T-T / A-A | 17.66±0.3 | 22.99±0.1 |
|  |  | 5’ CAGT | C-C / G-G | 17.45±0.5 | 22.64±0.5 |
|  |  | 5’ CACA | C-C / A-A | 14.48±0.4 | 20.22±0.5 |
|  | 5’ GACT | 5’ TCTC | C-C / T-T | 14.28±0.2 | 10.14±0.4 |
|  |  | 5’ ACAC | A-A / C-C | 15.30±0.1 | 10.56±0.2 |
|  |  | 5’ AGAG | G-G / A-A | 13.54±1.0 | 16.76±0.5 |
|  |  | 5’ TGAC | A-A / T-T | 12.24±1.1 | 9.60±0.1 |
|  |  | 5’ ACTG | G-G / C-C | 8.58±0.2 | 10.91±0.1 |
|  |  | 5’ TGTG | G-G / T-T | 10.37±0.2 | 16.96±0.2 |
| **3 Base pairs** | 5’ AGTC | 5’ GTGA | T-T / G-G /A-A | - | - |
|  |  | 5’ CTGT | C-C / T-T /G-G | - | - |
|  |  | 5’ CAGA | C-C / G-G /A-A | - | - |
|  |  | 5’ CTCA | C-C / T-T /A-A | - | - |
|  | 5’ GACT | 5’ TCAC | A-A / C-C /T-T | - | - |
|  |  | 5’ ACAG | G-G / A-A /C-C | - | - |
|  |  | 5’ TCTG | G-G / C-C /T-T | - | - |
|  |  | 5’ TGAG | T-T / G-G /A-A | - | - |
| **4 Base pairs** | 5’ AGTC | 5’ CTGA | C-C / T-T  G-G / A-A | - | - |
|  | 5’ GACT | 5’ TCAG | G-G / A-A  C-C / T-T | - | - |

**
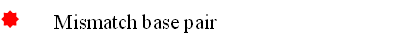
**


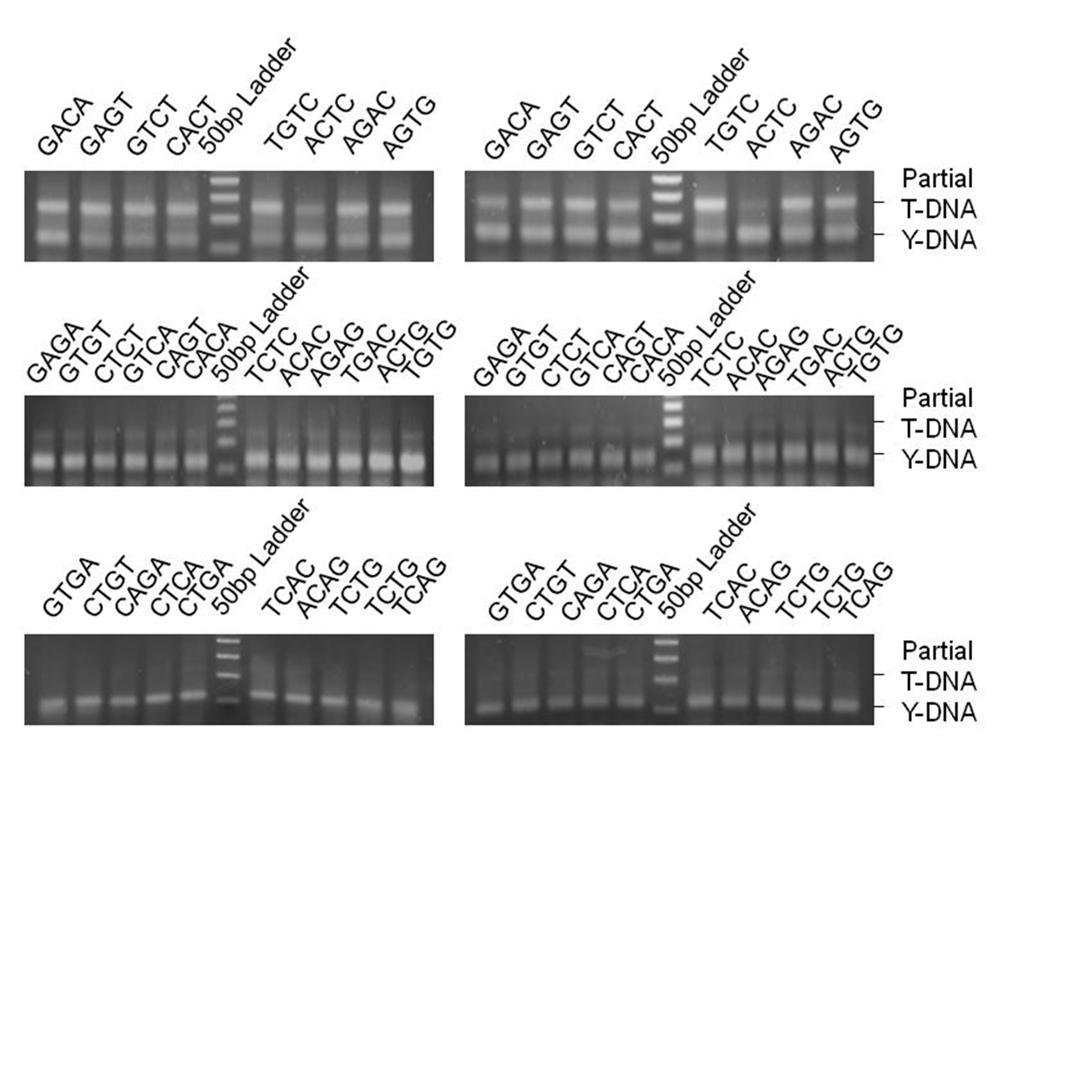


**Figure S6. Formation of Loop T-DNA.** Using GACT and GAGT overhang sequences with reasonable ligation efficiencies, LT-DNA, which can capture RNA markers in a cancerous cell, was assembled. Initially, LT-DNA was made from a single L-DNA, which can detect target EZH2 mRNAs. After the formation of LT-DNA, the same molar concentration of target DNA complimentary to the EZH2 mRNA sequence and to the loop sequence of LT-DNA was added to the LT-DNA solution at 37°C for 4 hours. LT-DNA effectively diagnosed specific mRNA markers, and the DNA-RNA binding affinity was similar that of DNA-DNA depending on the sequence^8,9^.


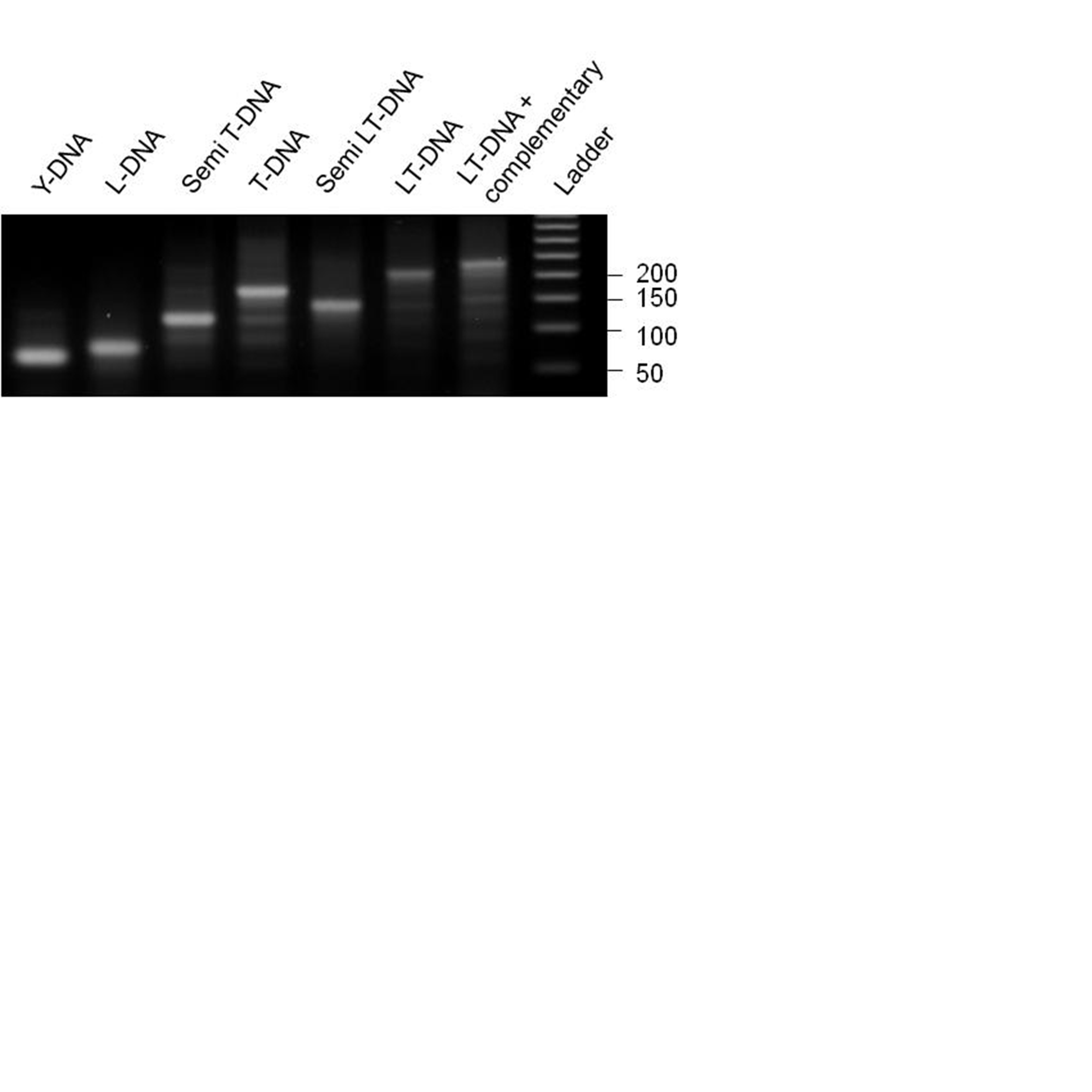


**Table S1.** **Preparation of oligonucleotides for T-DNA blocks.** Sequence information of T-DNA block. Subscript W, E and C mean WY, EY and CY.

| **Name** | **5’-end** | **sequence** |
| --- | --- | --- |
| Y_C1_ | - | TGG ATC CGC ATG ACA TTC GCC GTA AG-3' |
| Y_C2_ | 5'/Phos/GACT | CTT ACG GCG AAT GAC CGA ATC AGC CT-3' |
| Y_C3_ | 5'/Phos/GCAT | AGG CTG ATT CGG TTC ATG CGG ATC CA-3' |
| Y_W1_ | - | TGG ATC CGC ATG ACA TTC GCC GTA AG-3' |
| Y_W2_ | - | CTT ACG GCG AAT GAC CGA ATC AGC CT-3' |
| Y_W3_ | 5'/Phos/AGTC | AGG CTG ATT CGG TTC ATG CGG ATC CA-3' |
| Y_E1_ | - | TGG ATC CGC ATG ACA TTC GCC GTA AG-3' |
| Y_E2_ | - | CTT ACG GCG AAT GAC CGA ATC AGC CT-3' |
| Y_E3_ | 5'/Phos/ATGC | AGG CTG ATT CGG TTC ATG CGG ATC CA-3' |

**Table S2.** **Preparation of oligonucleotides for LT-DNA blocks.** (a) Sequence information of LT-DNA block. (b) Sequence of multi-functional LT-DNA block

a

| **Name** | **5’-end** | **sequence** |
| --- | --- | --- |
| Y_C1_ | - | TGG ATC CGC ATG ACA TTC GCC GTA AG-3' |
| Y_C2_ | 5'/Phos/GACT | CTT ACG GCG AAT GAC CGA ATC AGC CT-3' |
| Y_C31_ | 5'/Phos/GAGT | AGG CTG ATT CGG TTC ATG CGG ATC CA-3' |
| Y_loop-mRNA_ | GCG AGG CCA GAC TGG GAA GA A ATC TGC TCGC | TGG ATC CGC ATG ACA TTC GCC GTA AG-3' |
| Y_W2_ | - | CTT ACG GCG AAT GAC CGA ATC AGC CT-3' |
| Y_W3_ | 5'/Phos/AGTC | AGG CTG ATT CGG TTC ATG CGG ATC CA-3' |
| Y_loop-mRNA_ | GCG AGT CAA CAT CAG TCT GAT AAG CTA CTCGC | TGG ATC CGC ATG ACA TTC GCC GTA AG-3' |
| Y_E2_ | - | CTT ACG GCG AAT GAC CGA ATC AGC CT-3' |
| Y_E31_ | 5'/Phos/ACTC | AGG CTG ATT CGG TTC ATG CGG ATC CA-3' |

b

| **Name** | **5’-end** | **sequence** |
| --- | --- | --- |
| Y_C1_ | - | TGG ATC CGC ATG ACA TTC GCC GTA AG-3' |
| Y_C2_ | 5'/Phos/GACT | CTT ACG GCG AAT GAC CGA ATC AGC CT-3' |
| Y_C31_ | 5'/Phos/GAGT | AGG CTG ATT CGG TTC ATG CGG ATC CA-3' |
| Y_loop-mRNA_ | 5IAbRQ/GCG AGG CCA GAC TGG GAA GA A ATC TGC TCGC/iCy5/ | TGG ATC CGC ATG ACA TTC GCC GTA AG-3' |
| Y_W2_ | - | CTT ACG GCG AAT GAC CGA ATC AGC CT-3' |
| Y_W3_ | 5'/Phos/AGTC | AGG CTG ATT CGG TTC ATG CGG ATC CA-3' |
| Y_loop-miRNA_ | 5IAbRQ/GCG AGT CAA CAT CAG TCT GAT AAG CTA CTCGC/iCy3/ | TGG ATC CGC ATG ACA TTC GCC GTA AG-3' |
| Y_E2_ | - | CTT ACG GCG AAT GAC CGA ATC AGC CT-3' |
| Y_E31_ | 5'/Phos/ACTC | AGG CTG ATT CGG TTC ATG CGG ATC CA-3' |

**Supplementary References**

1. Rajagopalan, M., Rahmouni, A. R. & Well, R. D. Flanking AT-rich tracts cause a structural distortion in Z-DNA in plasmids. *J Biol Chem* **265**, 17294-17299 (1990).
2. Vinogradov, A. E. DNA helix: the importance of being GC-rich. *Nucleic Acids Res* **31**, 1838-1844 (2003).
3. Luo, D., Cu, Y. T., Li, Y. & Um, S. H. DNA Vaccines : Methods and protocols, eds Saltzman, W. M., Shen, H., Brandsma, J. L. (Humana Press, New Jersey) Chapter 10 (2006).
4. Allawi, H. T., SantaLucia, J. Thermodynamics and NMR of internal G·T mismatches in DNA. *Biochemistry* **36**, 10581-10594 (1997).
5. Bommarito, S., Peyret, N. & SantaLucia, J. Thermodynamic parameters for DNA sequences with dangling ends. *Nucleic Acids Res* **28**, 1929-1934 (2000).
6. Breslauer, K. J., Frank, R., Blocker, H. & Marky, L. A. Predicting DNA duplex stability from the base sequence. *Proc Natl Acad Sci USA* **86**, 3746-3750 (1986).
7. Rohs, R., *et al*. The role of DNA shape in protein-DNA recognition. *Nature* **461**, 1248-1253 (2009).
8. Sugimoto, N., *et al*. Thermodynamic parameters to predict stability of RNA/DNA hybrid duplexes. *Biochemistry* **34**, 11211-11216 (1995).
9. Kankia, B. I., Marky, L. A. DNA, RNA, and DNA/RNA oligomer duplexes: a comparative study of their stability, heat, hydration, and Mg^2+^ binding properties. *J Phys Chem B* **103**, 8759-8767 (1999).
